# Bivalirudin Attenuates Thrombin-Induced Endothelial Hyperpermeability via S1P/S1PR2 Category: Original Articles

**DOI:** 10.3389/fphar.2021.721200

**Published:** 2021-08-03

**Authors:** Haowen Ye, Yizhi Zhang, Yihui Huang, Biao Li, Ruhao Cao, Libing Dai, Bin Huang, Pingge Tian, Li Li, Yaling Han

**Affiliations:** ^1^ Department of Cardiology, Guangzhou Red Cross Hospital, Jinan University, Guangzhou, China; ^2^ Shanghai Jiao Tong University School of Medicine, Shanghai, China; ^3^ Department of Pediatrics, Guangzhou Red Cross Hospital, Jinan University, Guangzhou, China; ^4^ Guangzhou Institute of Traumatic Surgery, Guangzhou Red Cross Hospital, Jinan University, Guangzhou, China; ^5^ Cardiovascular Research Institute and Department of Cardiology, General Hospital of Northern Theater Command, Shenyang, China

**Keywords:** bivalirudin, S1P, s1pr2, endothelial (dys)function, thrombin, permeabiilty

## Abstract

**Aims:** To explore the role of the Sphingosine 1-Phosphate (S1P)/Receptor2 (S1PR2) pathway in thrombin-induced hyperpermeability (TIP) and to test whether bivalirudin can reverse TIP via the S1P-S1PRs pathway.

**Methods and Results:** Using western blot, we demonstrated that Human umbilical vein endothelial cells (HUVECs) that were cultured with 2 U/ml thrombin showed significantly increased S1PR2 expression while S1PR1and three kept unchanged. Such increment was attenuated by JTE-013 pretreatment and by presence of bivalirudin. Exposure of 2 U/ml of thrombin brought a higher level of S1P both intracellularly and extracellularly within the HUVECs by using ELISA detecting. Thrombin induced S1P and S1PR2 increment was restored by usage of PF543 and bivalirudin. Bivalirudin alone did not influenced the level of S1P and S1PR1,2, and S1PR3 compare to control group. As a surrogate of cytoskeleton morphology, phalloidin staining and immunofluorescence imaging were used. Blurry cell edges and intercellular vacuoles or spaces were observed along thrombin-exposed HUVECs. Presence of JTE-013 and bivalirudin attenuated such thrombin-induced permeability morphological change and presence of heparin failed to show the protective effect. Transwell chamber assay and probe assay were used to measure and compare endothelial permeability *in vitro*. An increased TIP was observed in HUVECs cultured with thrombin, and coculture with bivalirudin, but not heparin, alleviated this increase. JTE-013 treatment yielded to similar TIP alleviating effect. *In vivo*, an Evans blue assay was used to test subcutaneous and organ microvascular permeability after the treatment of saline only, thrombin + saline, thrombin + bivalirudin, thrombin + heparin or thrombin + JTE-013. Increased subcutaneous and organ tissue permeability after thrombin treatment was observed in thrombin + saline and thrombin + heparin groups while treatment of bivalirudin and JTE-013 absent this effect.

**Conclusion:** S1P/S1PR2 mediates TIP by impairing vascular endothelial barrier function. Unlike heparin, bivalirudin effectively blocked TIP by inhibiting the thrombin-induced S1P increment and S1PR2 expression, suggesting the novel endothelial protective effect of bivalirudin under pathological procoagulant circumstance.

## Introduction

The effect of increased permeability by thrombin has been shown, and thrombin-induced permeability (TIP) is a well-characterized and widely used barrier-disruptive effect used in a permeability model in endothelial cells (ECs) (Li et al., 2019; Abe et al., 2020). Thrombin affects cell morphology and intracellular contraction by reorganization of the cytoskeleton (Amerongen et al., 2000) and thus enhances EC paracellular permeability. Sphingosine 1-phosphate (S1P), a bioactive lipid produced and stored in platelets and released from activated platelets during blood coagulation activation, is critical and even indispensable for ECS barrier function (Burg et al., 2018). S1P regulates endothelial barrier function by activating various receptors. For example, activated S1PR1 and S1PR3 is associated with a protective barrier effect (Sanna et al., 2006), whereas the activation of S1P receptor 2 (S1PR2) destabilizes this effect (Sanchez et al., 2007). Recently, the balance between S1PR1 and S1PR2 signaling in a specific vascular bed was reported to determine endothelial responses to S1P *in vivo* (Lee et al., 2009; Chen et al., 2015; Marmonti et al., 2020)^.^ Crucially, the effect of S1P on vascular permeability strongly depends on its concentration. Studies have demonstrated that under physiological conditions, S1P acts as a barrier-protective agent, while at higher concentrations, S1P causes endothelial barrier disruption (Jozefczuk et al., 2020). S1P at physiological concentrations was recently shown to preserve endothelial barrier function by binding S1PR1, while excessive S1P induces endothelial malfunction by activating S1PR2, causing destruction to F-actin and tight junctions (Li et al., 2015), which might be the molecular mechanism by which the presence of S1P at low and high concentrations leads to opposite effects on barrier function. S1PR1 was revealed to be involved in and to enhance barrier function through thrombin at physiological concentrations in an autocrine or paracrine manner (Feistritzer and Riewald, 2005). Since abundant S1P is stored in blood platelets, S1P is released during blood coagulation activation (Yatomi et al., 2000), and thrombin can increase S1P generation by inducing the expression of SphK1 (Jozefczuk et al., 2020), it is conceivable that S1P is increased as a result of thrombin-mediated platelet activation during coagulation. Combined with the observation that thrombin has a barrier-disruptive effect at higher concentrations but is protective at lower concentrations (Vu et al., 1991), these findings led us to hypothesize that thrombin at a high concentration impairs EC barrier function by activating S1PR2. However, to date, the role of the S1PR1/2 balance and that of S1PR2 in TIP have been poorly understood.

Bivalirudin, a newly emerged direct-acting thrombin inhibitor, is a synthetic analog of hirudin. Previous studies have shown that hirudin can inhibit increased EC permeability (Chen et al., 2020); however, hirudin has not been clinically recommended because it has not been available in adequate amounts. Increasing new evidence has suggested that patients treated with bivalirudin show reduced rates of hemorrhage compared to those treated with unfractionated heparin in many procedures in which anticoagulation is needed, such as transradial coronary interventions (Kheiri et al., 2020), emergency percutaneous coronary intervention (PCI) for acute myocardium infarction (Han et al., 2015), especially emergency interventional therapy for elderly individuals (Meng et al., 2020), implantation of a left ventricular assist device (Bates et al., 2020) and cardiac surgery in children (Goswami et al., 2020)^.^ There are currently no reports on whether bivalirudin can inhibit the changes in EC permeability induced by thrombin. Our study focused on this gap, and we looked for evidence of bivalirudin’s protective effect against the thrombin-induced increase in endothelial permeability. As the breakdown of endothelial barrier integrity is associated with hemorrhagic events and thrombin increases endothelial permeability, we hypothesized that bivalirudin protects the endothelial barrier from TIP by inhibiting thrombin-induced changes in the cytoskeleton via the S1P/R pathway.

## Methods

### Western Blot Analysis

Human Umbilical Vein Endothelial Cells (HUVECs) (Procell Life Science and Technology co., Ltd. Wuhan, China) were washed with cold PBS. Then, we used RIPA lysis buffer to extract proteins, which were separated by 10% SDS-PAGE and transferred to PVDF membranes. Nonfat milk (5%) was used to block the membranes. Subsequently, different primary antibodies were bound to target proteins by incubation at 4°C overnight. Then, the membranes were incubated with HRP-linked secondary antibodies at room temperature for 2 h. Antibodies specific for s1pr1 and S1PR2 were used (Assay Biotech; United States).

### ELISA Assay of S1P in HUVECs

A human S1P ELISA kit (RJ11131, Shanghai Renjie Biotechnology Co., Ltd, Shanghai, China) is used to measure concentration of S1P following users’ guide supported by the manufacturer. In brief, after loading 50 μL of diluted sample (5x) and standard S1P reagent in test wells, the test plate is incubated at 37°C for 1 h, followed by five time washes. Enzyme-labeled antibody then is added as recommended concentration into each well. Incubate the plate at previously described condition. Chromogen solutions are added after washing the plate 5 times. Within 15 mins after a stop wash, optical density is measured at 450 nm (OD450) in a fluorescence microplate reader (Rayto RT-6100, Rayto Life and Analytical Sciences Co.,Ltd, Shenzhen, China). The concentrations of S1P in test samples are obtained from a standard curve.

### Measurement of Endothelial Permeability *in vitro*

The response of endothelial monolayer permeability to fluorescein isothiocyanate-conjugated 70-kDa dextran (FITC-dextran; Sigma-Aldrich Chemicals, St. Louis, MO, United States) was assessed using Transwell permeable membranes (12-well cell culture inserts) with a 0.4-μm pore size (Costar, Corning, NY, United States). HUVECs (1 × 10^5^) were seeded on gelatin-coated Transwell filters and allowed to grow after relevant stimulation. After incubation at 37°C for 3 days, samples from both the upper and lower chambers were collected for fluorometric analysis. Fluorescence intensity (FI) was measured using a microplate fluorescence reader (BioTek Inc., Winooski, VT, United States) with filter settings of 485 nm (excitation) and 538 nm (emission). Then, these fluorescence readings were used to calculate the permeability coefficient, which is indicative of vascular barrier disruption, with the following formula: permeability coefficient = FI (lower chamber) × 100%/[FI (upper chamber) + FI (lower chamber)].

### Immunofluorescence Staining

HUVECs were cultured on gelatinized coverslips at 1 × 10^5^ cells per coverslip in DMEM with 10% FBS and 1% penicillin for 2 days. After the required treatment, all HUVECs were fixed with 4% paraformaldehyde for 15 min, washed with PBS 3 times and permeabilized with 0.2% Triton X-100 at room temperature for 20 min. Then, the HUVECs were blocked with PBS containing 5% BSA at room temperature for 1 h. The HUVECs were then incubated with either PBS containing phalloidin (1 drop per milliliter) for 30 min at room temperature or primary antibodies against *β*-catenin (1:500) (CST, 8,480, United States), F-actin and Myosin IIa overnight at 4°C and secondary antibody (1:500) in the dark for 1 h at 37°C, followed by gelatin sectioning. The cells were visualized by laser scanning confocal microscopy (LSM710, Carl Zeiss, Germany).

### Measurement of Microvascular Permeability *in vivo*

Albumin extravasation was visualized with the Miles assay by measure the extravasation of Evans blue dye as previously described (Brash et al., 2018). 2% Evans blue (100 µl) was intravenously injected into the tail veins of adult male KM mice (20–25 g) followed by the corresponding treatment after 30 min: thrombin (Sigma; United States), JTE-013 (Sigma-Aldrich, J4080, United States), bivalirudin (Shenzhen Xinlitai Company, Shenzhen, China), heparin (Sciprogen, Shenzhen, China) and saline were subcutaneously injected into the back skin. Ten minutes after the treatments, all mice were anesthetized by an intraperitoneal injection of 25 mg/kg sodium pentobarbital and were sacrificed. Circular patches of skin from the backs of the mice were incubated in formamide for 1 h, and subcutaneous permeability was assessed under a stereomicroscope (Nikon SMZ25/SMZ18, Japan). Organ vascular permeability was assayed as previously described (Wick et al., 2018)**.** Different from subcutaneous detection, thrombin, bivalirudin, JTE-013 and saline was injected trans tail vein after Evans blue administration. The organs (heart, lung, liver, and kidney) were isolated by autopsy after sacrifice. The absorbance value at 600 nm was measured by using an absorbance plate reader (BioTek Inc., United States) and the 600 nm OD value/organ weight was calculated.

### Animal Experimental Design and Ethics Statement

All animal procedures had been approved by the Institutional Animal Care and Use Committee of the Medical University of Jinan University and were conducted in accordance with the Association for Assessment and Accreditation of Laboratory Animal Care (AAALAC). We performed all experimental procedures according to Guide for the Care and Use of Laboratory Animals Eighth Edition. The animals were housed in a temperature-controlled animal facility under a 12/12-h light/dark cycle in plastic cages with soft bedding and given free access to food and water.

### Statistical Analyses

Data are expressed as the mean ± SEM. Groups were statistically compared by *t* test or one-way analysis of variance. Statistical analyses were performed using SPSS 13.0 software (SPSS Inc., Chicago, IL, United States). Data are reported as the mean ± standard error of the mean. For comparisons of two conditions, Student’s t test was used; for comparisons of >2 conditions, 1-way ANOVA with Tukey’s post hoc test or repeated measures ANOVA with Bonferroni’s post hoc test was used when appropriate, as indicated in the figure legends. A *p* value <0.05 indicated statistical significance.

## Results

### Thrombin Induced S1PR2 Expression Increment, but not That of S1PR1 or S1PR3

**FIGURE 1:**
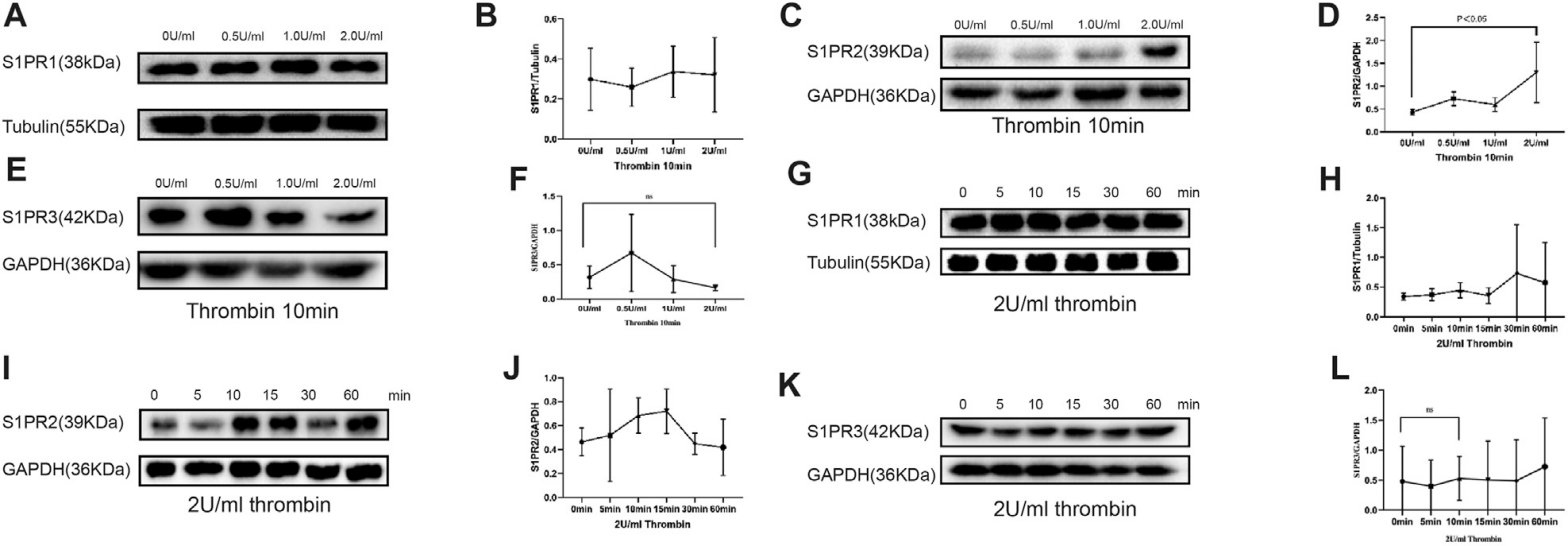
Compared with control (0 U/ml) group, S1PR1 and S1PR3 expression in HUVECs showed no significant change in groups of 0.5, 1, and 2 U/ml of thrombin exposure **(A, B, E, F)**, but S1PR2 expression was significantly elevated when HUVECs were treated with 2 U/ml thrombin compare to control group and low-dose thrombin treatment group **(C, D)** (*p* < 0.005). HUVECs cultured with 2.0 U/ml thrombin for 60 min did not show significant change in S1PR1 or S1PR3 level **(G, H, K, L)**, whereas the S1PR2 level was significantly increased after 10 min of 0.5 U thrombin treatment and peaked at 15 min compared with that in the control group **(I, J)**. The *p* value is calculated using the Student’s *t* test.

To test the effect of thrombin on S1PRs that are previously proved to be associated with endothelial barrier function, we cultured HUVECs with thrombin at 0 U/ml (control group), 0.5, 1.0, and 2.0 U/ml for 10 min. Western blotting was used to measure the protein levels of S1PR1, 2, and 3. Compared to those in the control group, S1PR1 and S1PR3 levels among cells cultured with thrombin at different concentrations were unchanged, while S1PR2 levels were significant increased upon culture with thrombin at only 2 U/ml (Figures 1A–F).

We then observed the time-dependent effect of thrombin on S1PRs. HUVECs were cultured with thrombin at 2 U/ml for 5, 10, 15, 30 and 60 min. The levels of S1PRs at different time points were then measured by western blot. S1PR1 and S1PR3 levels remained stable across all time points and were no different from those in the control group (Figures 1G,H,K,L). However, S1PR2 expression was significantly increased at 10 and 15 min compared with that in the control group (Figures 1I,J).

Hence, treatment with 2 U/ml thrombin for 10 min was used for the following experiment.

### Bivalirudin Inhibits the Thrombin-Induced Increase in S1P and S1PR2 Expression

**FIGURE 2:**
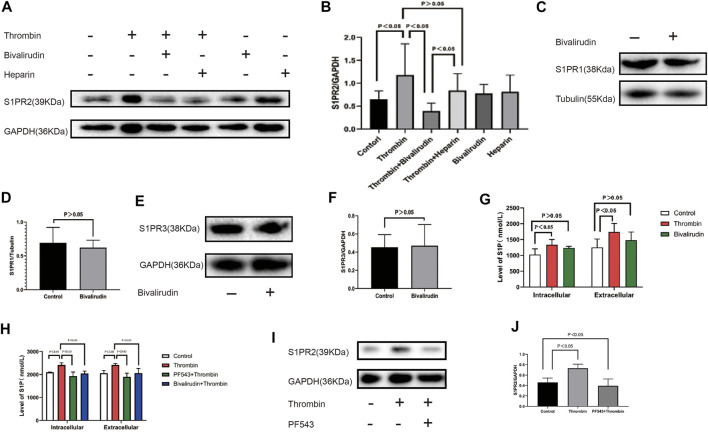
**(A)**: From left to right, the HUVECs were cultured under six conditions: control, 2 U/ml thrombin, 2 U/ml thrombin +30 μg/ml bivalirudin, 2 U/ml thrombin+4 U/ml heparin, 30 μg/ml bivalirudin only, and 4 U/ml heparin. Compared with the thrombin-treated group, HUVECs that were cultured with thrombin + bivalirudin exhibited lower S1PR2 expression, but HUVECs cultured with thrombin + heparin failed to show this change **(B)**. Bivalirudin alone did not influenced S1PR1 or S1PR3 expression **(C–F)**. Compared with control group, thrombin lead to intracellular and extracellular S1P level increment in HUVECs, while bivalirudin did not have such effect **(G)**. Usage of PF543, a potent SPHK1 inhibitor, could significantly reduce intracellular and extracellular S1P level compared with thrombin treatment alone in HUVECs and bivalirudin showed the similar effect as PF543 **(H)**. S1PR2 expression reduction was also observed with the presence of PF543 and bivalirudin **(I, J)**. The *p* value is calculated using the Student’s *t* test.

HUVECs were cultured under six conditions: saline (control group), 2 U/ml thrombin, 2 U/ml thrombin + bivalirudin (30 μg/ml), 2 U/ml thrombin + heparin (4 U/ml), bivalirudin alone and heparin alone. We found that compared to the control treatment, the use of thrombin alone significantly increased the S1PR2 level, and cells cultured with thrombin + bivalirudin showed lower S1PR2 expression, but cells cultured with thrombin + heparin failed to show this change. In the groups treated with bivalirudin and heparin alone, S1PR2 levels did not show a significant change compared with those in the control group, which verified that the reduction in S1PR2 in the thrombin + bivalirudin group did not result from a direct inhibitory effect of bivalirudin on S1PR2 (Figures 2A, B). To detect the effect of bivalirudin to S1PR1 and S1PR3, we measured S1PR1 and S1PR3 expression and noticed that neither of them changed significantly under bivalirudin exposure (Figures 2C, D, E, F). Next, *in vivo*, we detected the level of S1P, the ligand of S1PRs, by ELISA. Exposure of 2U/ml of thrombin brought a higher level of S1P both intracellularly and extracellularly within the HUVECs culture environment, while exposure of bivalirudin did not alter S1P level in neither of the sites (Figure 2G). To demonstrate the essential role of S1P in thrombin induced S1PR2 regulation, we then pre-treated HUVECs with an Sphk-1 inhibitor, which can inhibit S1P synthesis, and we found that thrombin failed to increase the level of S1P (Figure2H) and S1PR2 any more (Figure 2I, J). These findings indicate that bivalirudin treatment significantly abrogated the thrombin-induced S1PR2 increase in HUVECs and such effect is mediated by thrombin induced S1P synthesis.

### The Thrombin-S1P-S1PR2 Pathway Mediates Thrombin-Induced Permeability Increment and can Be Inhibited by Bivalirudin

**FIGURE 3:**
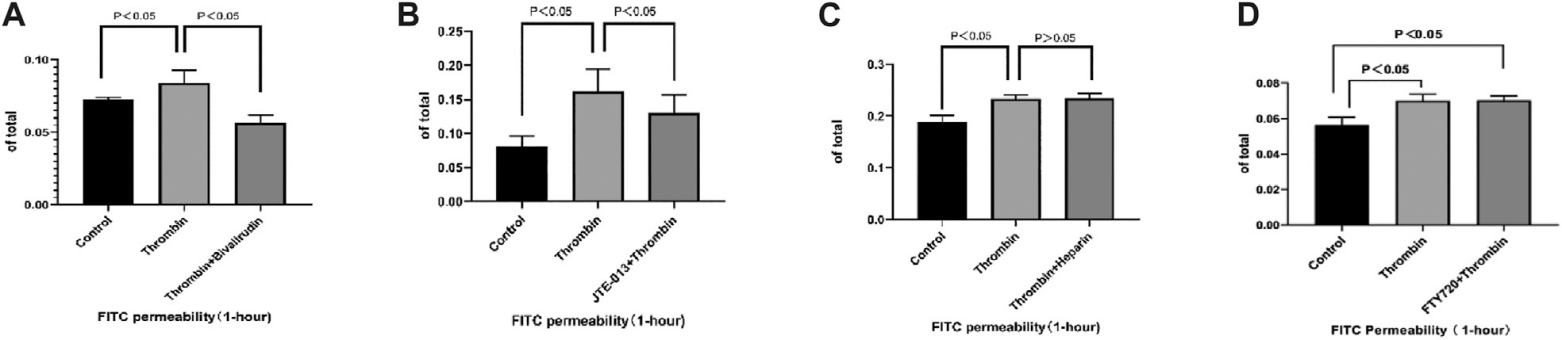
Compared with control group, treatment with thrombin lead to FITC permeability increment in HUVECs while use of bivalirudin blocked such effect **(A)**. Administration of JTE-013 shares similar blockage effect **(B)**. Co-treatment with FTY720 or heparin could not prevent FITC permeability increment caused by thrombin **(C, D)**. The *p* value is calculated using the Student’s *t* test.

To measure endothelial permeability, an *in vitro* FITC-dextran-based permeability assay kit was used. JTE-013 was used as a specific antagonist of S1PR2 HUVEC. Samples were collected for fluorometric analysis after incubation with saline, thrombin, or thrombin + JTE-013 for 10 min. We found that treatment of a confluent monolayer of HUVECs with thrombin led to a rapid increase in permeability compared with the permeability of the control group (Figure 3A). This increase was absent in the group treated with bivalirudin and JTE-013 plus thrombin (Figures 3A, B). Coincubation of heparin cannot yield to the restoration (Figure 3C). To rule out other S1PRs in TIP, we used FTY720, a potent antagonist of S1PR1, 3, 4, and 5, to treat HUVECs together with thrombin. There was no significant endothelial permeability change in FTY720 + thrombin group compared with that of thrombin alone treatment group (Figure 3D). This observation demonstrated the protective effect of bivalirudin on TIP and validated the specify role of S1PR2 in it.

### Bivalirudin Could Reverse S1PR2-Induced Cytoskeletal Changes Upon Thrombin Exposure

**FIGURE 4:**
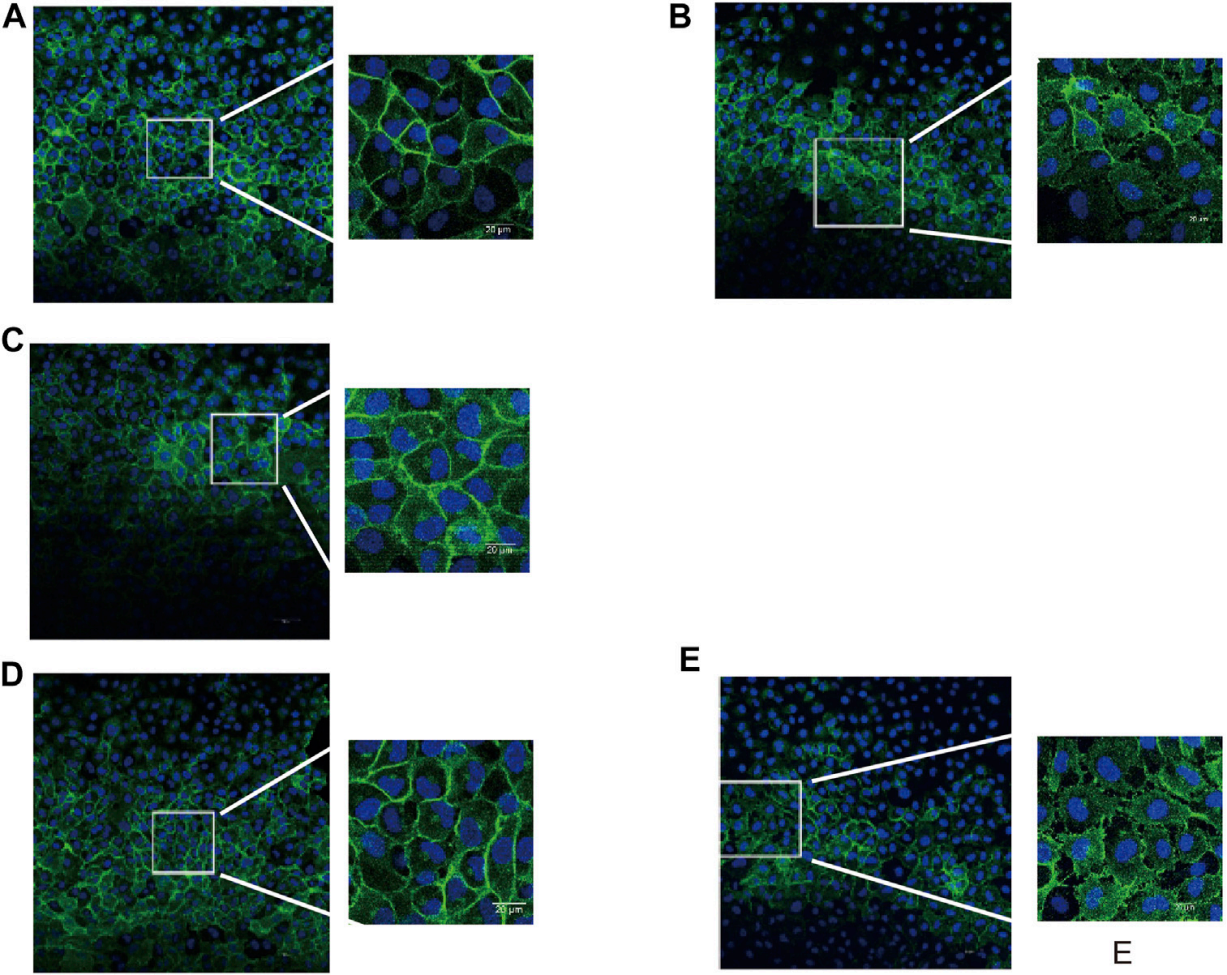
Effect of S1PR2 inhibitor or bivalirudin or heparin on *β*-catenin staining in HUVECs stimulated by thrombin. About 30 cells were selected from each group and observed. **(A)** Control group: cell profile was normal and cell-cell adjunction was tight **(B)**Thrombin treatment group: intercellular vesicle-like gaps appeared **(C)**and **(D)** Thrombin + JTE-013 group and thrombin + bivalirudin group: the morphological abnormality was restored as control group; **(E)** Thrombin + heparin group: The abnormal intercellular pattern as thrombin group preserved.

Maintaining a normal EC shape and tight cell-cell adherence junctions is essential for endothelial permeability. *β*-Catenin is an integral structural component among cadherin-based cell-cell adherents junction proteins. We carried out immunohistochemical staining for *β*-catenin and found that unlike the normal phenotype of the control group (Figure 4A), multiple intercellular vesicle-like gaps were observed between the thrombin-treated HUVECs (Figure 4B), which indicated that cellular connections were impaired. In both the thrombin + JTE-013 and thrombin + bivalirudin groups, this abnormal *β*-catenin phenotype was restored, and the phenotype was identical to that of the control group (Figures 4C, D). However, in the heparin + thrombin group, the intercellular vesicle-like gaps were maintained (Figure 4E).

**FIGURE 5A:**
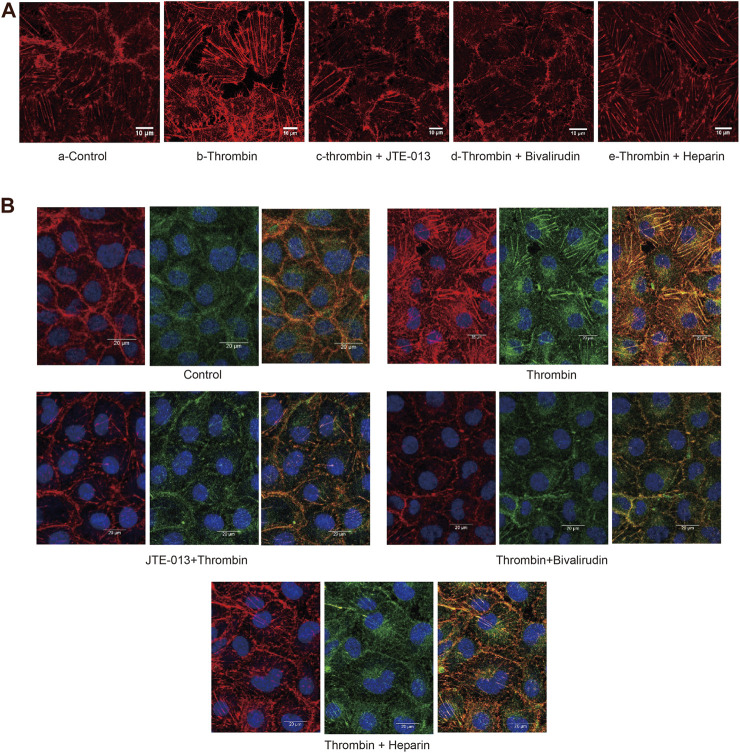
Effect of S1PR2 inhibitor or bivalirudin or heparin on F-actin staining in HUVECs stimulated by thrombin. About eight cells were selected from each group and observed. Compared to treatment **(A-a)**, thrombin treatment increased the density and thickness of F-actin, and a significant intercellular space was observed. Cells exhibited a gathered look and shrank **(A-b)**. The phenotype of the thrombin group was improved in the thrombin + JTE-013 and thrombin + bivalirudin groups **(A-c, d)**. In the heparin group, the cell edge was blurry. The majority of the F-actin detected showed obvious spaces **(A-e)**. Scale bar, 10 μm.

The F-actin protein forms the microfilaments of the cytoskeleton and plays an important role in cell shape. We then examined morphological changes in F-actin in HUVECs by phalloidin staining. We noticed an increase in F-actin accompanied by a “pull-away” intercellular phenotype, which resulted in a significant space between cells in the thrombin treatment group (Figure 5A-b) compared to those in the control group (Figure 5A-a). Less F-actin was observed in HUVECs in the thrombin + JTE-013 and thrombin + bivalirudin treatment groups, and the space between the cells was almost absent (Figure 5A-c,d). In the heparin treatment group, F-actin was clearly detected, and the space between cells was like that in the group treated with thrombin alone (Figure 5A-e).

In ECs, stress fibers are primarily composed of actin and myosin. We then stained for F-actin and myosin IIa under different conditions. In the control group, the edges of the HUVECs were sharp, and no intercellular gaps were observed (Figure 5B-a). In the group treated with thrombin only, a dramatic increase in polymerized F-actin and myosin IIa was observed, and the cell edge was blurry (Figure 5B-b). In the thrombin + JTE-013 and thrombin + bivalirudin groups, this increase was attenuated (Figures 5B-c,d), and polymerized F-actin and myosin IIa levels were the same as those in the control group. In the heparin group, no significant difference upon comparison with the group treated with thrombin alone was detected (Figure 5B-e).

These results indicated that thrombin impaired endothelial barrier function by injuring the cytoskeleton, but this impairment could be blocked by bivalirudin and JTE-013; however, heparin did not abrogate the thrombin-induced barrier impairment.

### The Protective Effect of Bivalirudin Against TIP Proceeds via S1PR2 *in vivo*

**FIGURE 6:**
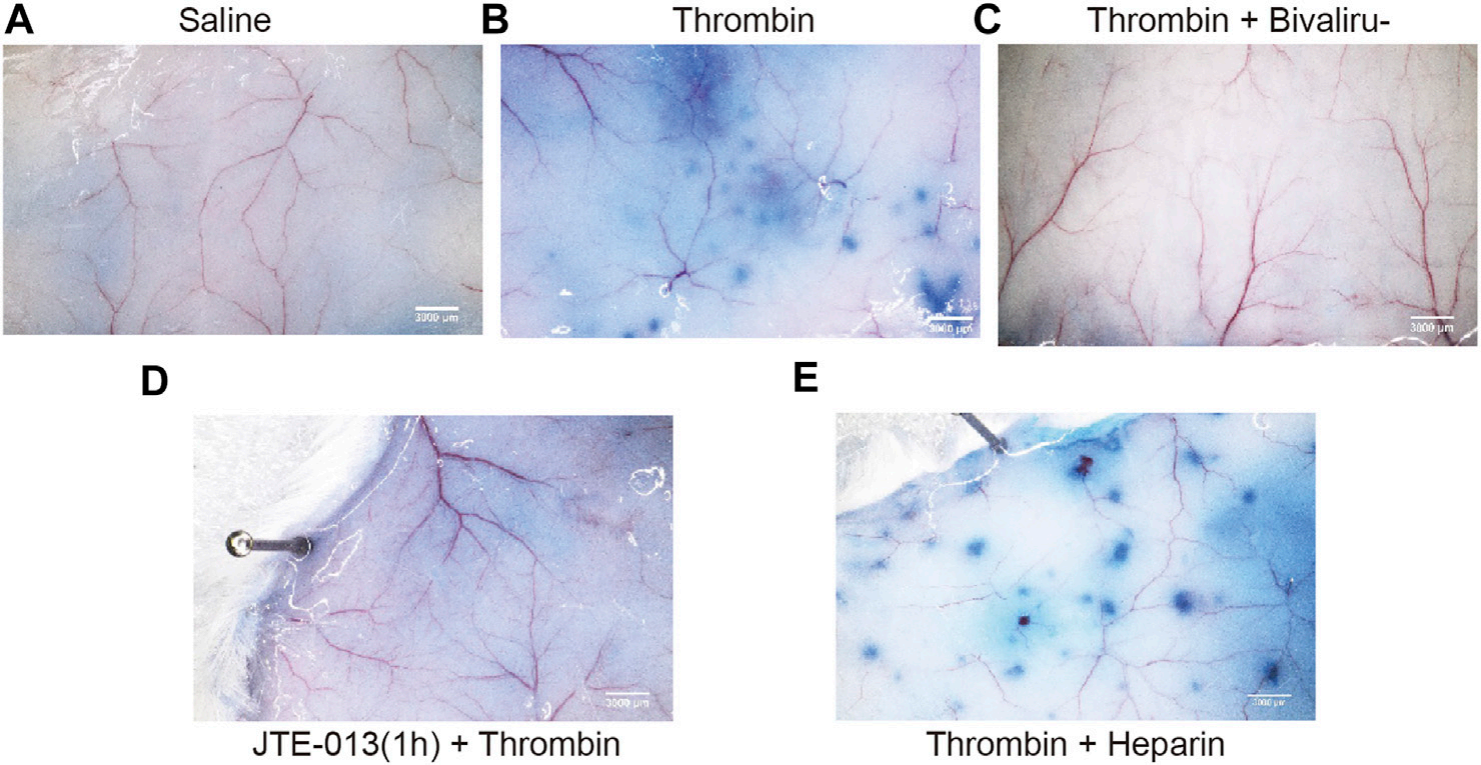
Changes of thrombin with or without JTE-013, bivalirudin and heparin were subcutaneously injected into the back skin on KM mice. No Evans blue exudation along the blood vessels was observed after saline injection in the tail vein (A). Ten minutes after thrombin injection, punctate Evans blue exudates were detected along the blood vessels (B). In the thrombin + bivalirudin group, subcutaneous Evans blue exudation was significantly increased compared with the in the group treated with thrombin alone (C), while in thrombin + heparin group, we noted a significant increase in Evans blue re-emerging along the blood vessels (D). However, when we pretreated cells with JTE-013, followed with thrombin injection, no significant Evans blue exudation along the blood vessels was observed (E).

An Evans blue extravasation assay was used to detect tissue microvascular permeability. The doses of bivalirudin, heparin and JTE-013 used were calculated based on the dose used in humans (FDA U.S. Department of Health and Human Services, 2005). At 30 min after Evans blue (100 μl) injection in the tail vein, the mice were randomly divided into five groups and treated as follows: subcutaneous saline injection as (control group); subcutaneous thrombin (100 U) injection (thrombin group); transvenous tail vein injection of JTE-013 (30 mg/kg), bivalirudin (9.3 mg/kg) and heparin (1230 U/kg) followed by subcutaneous thrombin (100 U) injection on the back (JTE-013 + thrombin group), bivalirudin and thrombin treatment (bivalirudin + thrombin group) and heparin and thrombin treatment (heparin + thrombin group). Ten minutes after saline, bivalirudin and heparin injection, the active clotting time (ACT) was measured with mouse retrobulbar venous blood to ensure that anticoagulation was effective. The average ACT was 65 s in the control group, 229 s in the bivalirudin group and 253 s in the heparin group. We found that the thrombin group (Figure 6B) showed significantly more Evans blue exudation along the blood vessels than the blank control group in the subcutaneous tissue (Figure 6A). Pretreatment with bivalirudin and JTE-013, followed by thrombin treatment, did not result in significant Evans blue exudation (Figure 6C, D), similar to the pattern observed in the control group. In the heparin pretreatment group, Evans blue exudation was revealed along blood vessels (Figure 6E), in the group treated with thrombin alone.

**FIGURE 7:**
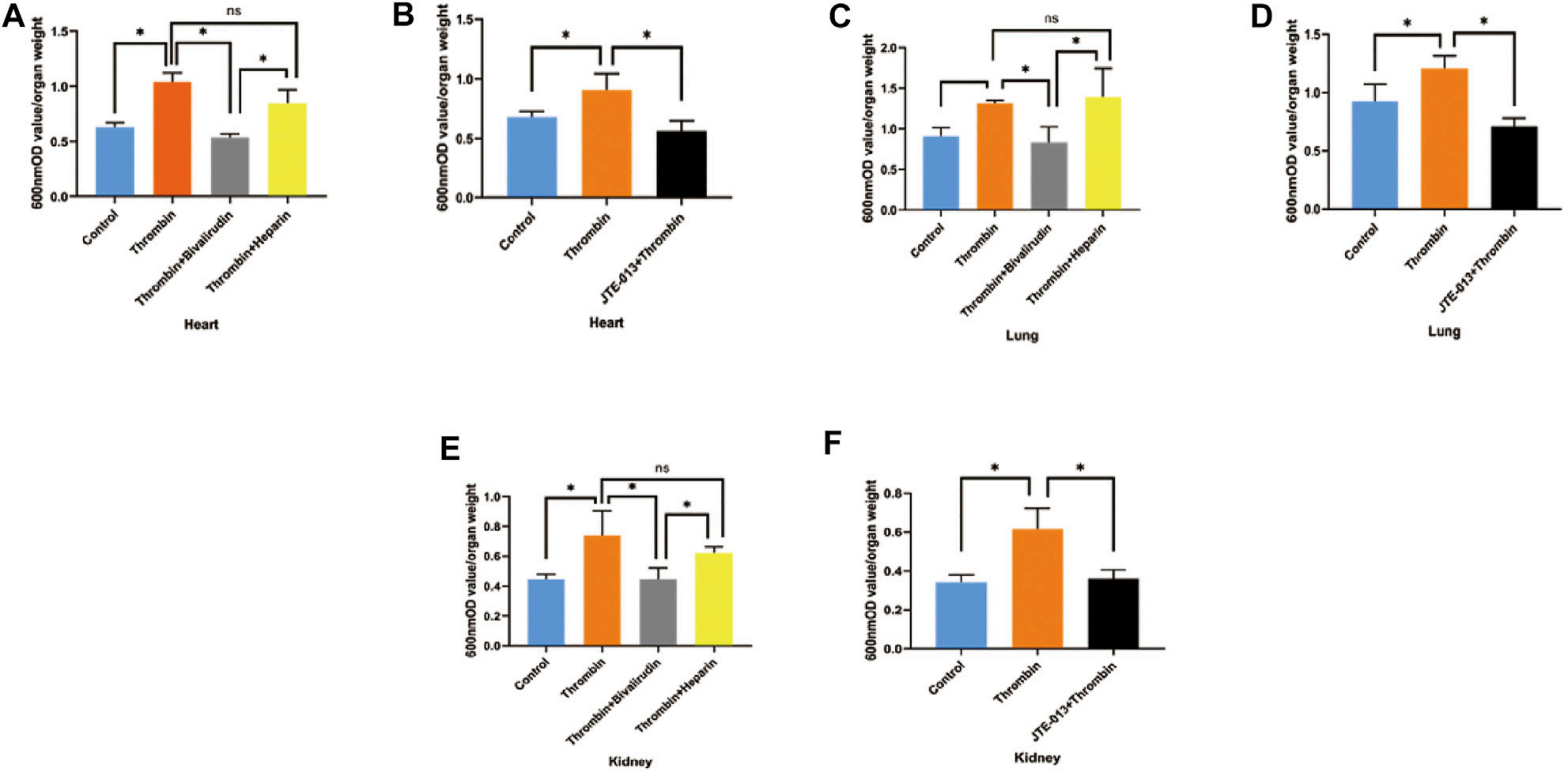
Changes of EB permeability in the organs of KM mice after intravenous injection of thrombin with or without JTE-013, bivalirudin and heparin. Thrombin treatment yielded significant increases in Evans blue exudation in tissues from the heart, lung and kidney. Treatment with bivalirudin and JTE-013 significantly reduced Evans blue exudation, but that observed upon heparin use did not significantly differ from that observed upon treatment with thrombin alone **(A, C,E)**. Compared with thrombin treatment, pretreatment with JTE-013 significantly reversed the thrombin-induced increase in Evans blue exudation **(B, D, F)**. The *p* value is calculated using the Student’s *t* test.

Tissue permeability of the heart, kidney and lung was assessed with the same protocol used to measure subcutaneous permeability, except thrombin was injected into the tail vein. As shown in Figure 7, we noticed increased Evans blue extravasation in the heart, lung and kidney samples of mice treated with thrombin compared with those in the control group, but cotreatment with bivalirudin significantly decreased Evans blue extravasation in tissues from all of these organs. Heparin use did not affect the increase in Evans blue extravasation observed in the group treated with thrombin alone (Figure 7A,C,E). In the JTE-013 pretreatment group, no difference in Evans blue staining in the heart, lung or kidney was observed compared with that in the control group (Figure 7B,D,F). We did not observe any changes in the liver upon the above treatments (Supplement), which may be due to the first-pass effect of the drugs.

## Discussion

### S1PR2 Mediates Thrombin-Induced Endothelial Permeability

#### Thrombin’s Concentration-Dependent Effect on S1PRs

Studies have demonstrated that under physiological conditions, S1P acts as an endothelial barrier-protective agent, while at higher concentrations, S1P causes endothelial barrier disruption (Komarova et al., 2007; Xiong and Hla, 2014). The underlying mechanism might be the different responses of S1PRs to different concentrations of S1P. Lower concentrations of S1P are previously reported to mainly activate S1PR1, while conversely, high concentrations of S1P activated S1PR2 (Marmonti et al., 2020). In our study, S1P1and three maintains stable in any concentration of thrombin stimulation. Findings regarding S1PR2 expression under stimulation of different concentrations of thrombin at from 0.5 to 2 U/ml and that 2 U/ml thrombin yield an increased S1P level confirmed the previous observation and further implied a relationship between thrombin-S1P-S1PR2 pathway and endothelial barrier function.

#### Time Based Response of S1PRs Under Thrombin Exposure

Previous findings have indicated that S1PR2 is upregulated at the early response phase of the endothelium after injury, as well as in the pro-adhesive and procoagulant phenotypes of the endothelium (Zhang et al., 2013)^.^ In our study, elevated S1PR2 expression was detected at 10–15 min after thrombin treatment, but S1PR1 and S1PR3 expression level did not change within 30 min under same levels of thrombin treatment.

#### Cytoskeleton Changes Caused by Thrombin-S1PR2 Pathway

Researchers have shown that thrombin increases endothelial permeability by inducing stress fiber formation and the disassembly of adherent junctions (Amerongen et al., 2000). In our study, the elevated formation of stress fibers was observed, intercellular gaps appeared and EC edges became indistinct after thrombin treatment. Staining of *β*-Catenin, which was mainly located at the cytoplasmic side of the membrane within cadherin-based cell-cell connections, also revealed a discontinuous cell edge and decreased intercellular connections. Again, pretreatment with JTE-013 blocked thrombin from exerting such effects. Our research strongly suggests that TIP is related to cytoskeletal regulation, consistent with a previous study, and that this regulation is mediated by the S1PR2 pathway (Sanchez et al., 2007).

#### S1PR2 is Critical to Thrombin-Induced Tissue Level Permeability Changes

Previous studies have demonstrated the thrombin-induced permeability increment within Transwell chamber *in vivo* and observed thrombin-induced organ permeability *in vitro* (Escue et al., 2017). Clinically, the causal role of thrombin in lung ischemia-reperfusion injury has been established (Farivar et al., 2006). A previous study demonstrated that transactivation of S1PR2 evoked barrier-disruptive responses in the lung, as shown by detecting the alleviated inflammatory responses of S1PR2−/− mice in lipopolysaccharide (LPS)-induced acute lung injury model compared with control group (Sammani et al., 2010). It is also well established that S1PR2’s significant role in Rho-ROCK-PTEN pathway in HUVECs (Sanchez et al., 2007). All these evidences suggest that thrombin-S1P-S1PR2 may play a critical role in TIP, which is our main hypothesis in this study.

*In vitro*, we verified the TIP could be triggered by thrombin induced S1P synthesis, which is also coincident with previous study (Mahajan-Thakur et al., 2015), and be restored by inhibition of S1PR2 while not by inhibition of S1PR1, S1PR3, S1PR4, and S1PR5. *In vivo*, we found that subcutaneous and trans tail venous administration of thrombin significantly increased subcutaneous and organic Evans blue exudation.

### Bivalirudin can Attenuate TIP Through the Thrombin-S1P-S1PR2 Pathway

In our study, we demonstrated that bivalirudin can block TIP through inhibition of thrombin induced S1P synthesis. There were no sound evidence suggesting direct effect of bivalirudin on S1P or on S1PR2. Exposure of bivalirudin only would not alter S1P synthesis, S1PR2 expression or FITC permeability *in vitro* and would not affect organic Evans blue leakage *in vivo*, which indicated that the potential protective perspective of bivalirudin was valid under pathological procoagulant circumstance only.

### Limitations

In this study, we try to disclosure the role of thrombin induced S1P/S1PR2 increment in TIP and the protective effect of bivalirudin both *in vitro* and *in vivo*. We interfered S1P synthesis and S1PR2 expression by using of PF432, the inhibitor of SPHK1, and JTE-013, the specific antagonist of S1PR2. However, specific downstream targets of S1P/S1PRs remains to be discovered. Appropriate methods of next generation sequencing analysis might be suitable approaches to screen out these targets and lead to further researches but because of the limitation of time and capacity we did not perform them in this study. In term of clinical benefits of bivalirudin, another limitation of this study is absence of a clinical cohort study or a randomized controlled trial, which might be conducted in the future.

## Data Availability

The original contributions presented in the study are included in the article/supplementary material, further inquiries can be directed to the corresponding authors.
